
JOURNAL INFORMATION
==============================
NLM Title Abbreviation: ScientificWorldJournal
Journal ID: ScientificWorldJournal
NLM Journal ID: 101131163
Journal ID: TSWJ

The Scientific World Journal

ISSN: 2356-6140
EISSN: 1537-744X
Publisher: Wiley

ARTICLE INFORMATION
==============================
PMCID: PMC6084037
PMID: 30147585
DOI: 10.1100/tsw.2001.128
Article ID: 491380
Article version: 1
Subjects: Short Report

CDNA Cloning, Heterologous Expression and Characterization of Anti-Neuroexcitation Peptides (ANEPS) of Scorpion Buthus martensii Karsch

Zhang Jing-Hai 1 2
Hua Zi-Chun 1 *
Zhu De-Xu 1
1 State Key Laboratory of Pharmaceutical Biotechnology, College of Life Sciences, Nanjing University, Nanjing 210093, China
2 Department of Biochemistry, Shenyang University of Pharmacy, Shenyang 110015, China
*Zi-Chun Hua: zchua@nju.edu.cn
Publication date: 2001
Electronic publication date: 2001 Dec 12
Volume: 1
First page: 106
Last page: 106
Copyright: Copyright © 2001 Jing-Hai Zhang et al.
Copyright year: 2001
License: This is an open access article distributed under the Creative Commons Attribution License, which permits unrestricted use, distribution, and reproduction in any medium, provided the original work is properly cited.
License URL: https://creativecommons.org/licenses/by/3.0/

==============================
Miami *Nature Biotechnology* Short Reports
*TheScientificWorld* (2001) 1(S3), 106SR
ISSN 1532-2246; DOI 10.1100/TSW.2001.128

### CDNA CLONING, HETEROLOGOUS EXPRESSION AND CHARACTERIZATION OF ANTI-NEUROEXCITATION PEPTIDES (ANEPS) OF SCORPION *BUTHUS MARTENSII* KARSCH

Jing-Hai Zhang[1,2], Zi-Chun Hua[1,*], De-Xu Zhu[1]

[1]State Key Laboratory of Pharmaceutical Biotechnology, College of Life Sciences, Nanjing University, Nanjing 210093, China;
[2]Department of Biochemistry, Shenyang University of Pharmacy, Shenyang 110015, China,
[*]zchua@nju.edu.cn

**INTRODUCTION.** The neurotoxins from scorpion venoms are specifically active on mammals or insects, and divided into two main categories based on their molecular sizes and pharmacological activities. The short-chain toxins consist of 30 to 40 amino acid residues with 3 or 4 disulfide bonds and predominantly act upon potassium or chloride channels, meanwhile the toxins contain 60 to 70 amino acid residues, the long-chain toxins, with 4 disulfide bonds primarily influence sodium channels. These toxins can selectively bind to the corresponding channels on the membrane of excitable cells, impair the initial rapid depolarization phase of the action potential in nerve and muscle, thus result in neurotoxicity. In China, scorpions and their tissues or extracts have been extensively used for treatment of apoplexy, epilepsy, convulsion, facial paralysis, hemiplegia and anti-inflammation etc in traditional Chinese medicine for over thousands of years. Neurotoxins of the scorpion *Buthus martensii* Karsch (Bmk), a representative species in China and east Asia, have been widely studied in recent fifteen years, including studies on their primary and three-dimensional structures, their cDNA and genomic genes, their physiological and biological functions, and their pharmaceutical properties. We cloned a new anti-neuroexcitation peptide cDNA, expressed and purified it in E. coli and characterized its biological activities. The anti-neuroexcitation peptide is highly homologous to the previously reported anti-epilepsy peptide and shows strong calcium channel enhancement activity.

**METHOD.** According to the previously reported N-terminal amino acid sequence of anti-epilepsy peptide (1), two degenerate primers were designed and synthesized. Total RNA was isolated from scorpion telsons and reverse transcribed. RACEs were performed. The PCR product was cloned and sequenced. The putative mature anti-neuroexcitation peptide (ANEP) encoding cDNA was then cloned into pET28a expression plasmid. Recombinant ANEP was purified by His-Bind affinity column. The resulting elute was desalted and concentrated with Amicon stirred ultrafiltration cells (1kDa membrane). Then the protein was further purified on Superdex 30 chromatography and analyzed by SDS-PAGE. Anti-neuroexcitation bioassay was performed essentially according to Xu et al (2) by using thiosemicarbazide induced mice convulsion model. Physiologic saline was used as blank control, sodium phenobarbital (4.0mg/kg) as positive control and recombinant anti-neuroexcitation peptide (2.0mg/kg) as sample respectively. The delayed time on the seizure of convulsive symptom in ANEP group and positive group, in comparison with the control group, was expressed in percentage of the control group and was used as indicator of anti-neuroexcitation biological activity. The cDNA sequence has been submitted to GenBank under accession number AF122003.

**RESULTS**. Anti-neuroexcitation peptide (ANEP) totally has 82 amino acids, and the first 20 amino acids are supposed to be signal peptide. There are eight cysteines, about 12%, in ANEP. It is homologous to anti-epilepsy peptide, 63% similarity in the corresponding amino acid sequence, but have obvious differences in amino acid sequence. The cDNA encoding mature scorpion anti-neuroexcitation peptides was cloned into expression plasmid pET28a, fused to six consecutive histidines at its C-terminal. Recombinant ANEP was expressed upon IPTG induction and was about 15% of total cellular proteins, with most products aggregated in the insoluble inclusion body form. After sonication, the supernatant fraction was passed over the $Ni^{2+}$-His-Bond column, and eluted by 1M imidazole, 0.5M NaCl, 20mM Tris-HCl, pH7.9. The elution fraction was desalted by ultra-filtration, further purified with Superdex 30 column. Recombinant ANEP was purified to homogeneity with the purity over 95% analyzed by SDS-PAGE. The overall recovery yield from the supernatant fraction after two-step chromatography was 0.7%. About 3mg pure recombinant ANEP-Ile-Glu-$His_6$ was obtained from 2 liters of flask culture. The anti-neuroexcitation activity of purified ANEP-Ile-Glu-$His_6$ was assayed on the animal model of convulsion induced by thiosemicarbazide. Comparing with the saline control, 2.0mg/kg of recombinant ANEP could display 18.0% delay in the convulsive seizure ($p<0.01$) and thus showed distinct anti-neuroexcitation biological activity. ANEP also has positive impact on Calcium channels in CNS neurons. The peptide-induced enhancement of Calcium currents is obvious and reversible. Further study is going one to investigate its biochemical, biophysical and pharmaceutical properties and the potentials as therapeutic peptide.

**REFERENCES.**

1. Zhou, X.H., Yang, D., Zhang, J.H., Liu, C.M., and Lei, K.J. (1989) Biochem. J. 257, 509-517
2. Xu, S.Y., Bian, R.L., and Chen, X. (1991) Experimental Methods of Pharmacology, People's Hygiene Press, Beijing, China
